# Interrelatedness of Family and Parenting Risk Factors for Juvenile Delinquency: A Network Study in U.S. and Dutch Juveniles

**DOI:** 10.1177/0306624X241240697

**Published:** 2024-04-02

**Authors:** Claudia E. van der Put, Mark Assink

**Affiliations:** 1 University of Amsterdam, The Netherlands

**Keywords:** risk factor, criminal behavior, delinquency, juvenile, network analysis

## Abstract

Family interventions that address a diversity of family and parenting factors are often used to prevent juvenile delinquency, but are effective to only a limited extent. This study applied a network approach to risk factors for juvenile delinquency and examined the interrelatedness of specifically family and parenting risk factors in a U.S. and separate Dutch sample of juveniles and their family members. Differences in interrelatedness between these samples were examined as well. Secondary analyses were conducted on data collected in the United States with the Washington State Juvenile Court Assessment (WSJCA) and on data collected in the Netherlands with a Dutch-adapted translation of the WSJCA. Network analyses were performed, separately for the U.S. (*N* = 13,613) and Dutch (*N* = 3,630) sample, on seven risk factors that were assessed with a three-point Likert scale ranging from each factor’s protective side to a corresponding risk side. In the U.S. sample network, “inadequate parental punishment” and “lack of parental supervision” that both refer to an authoritarian parenting style were the most “central” factors and had the strongest associations with the other risk factors. In the Dutch sample network, “the family not providing opportunities” and “inadequate parental reward” were the most “central” factors, which refer to an authoritative parenting style. The family and parenting factors identified as most central in the networks may be promising to address in family interventions, as it can be expected that both the directly addressed problems and their correlated problems will improve. The current results may inform attempts to strengthen family interventions for juvenile delinquency in the United States and the Netherlands.

## Introduction

Family interventions are often used to prevent juvenile delinquency and aim to address a diversity of parenting factors, such as a lack of parental supervision and insufficient parental involvement. However, meta-analyses have shown that these interventions are effective to only a limited extent (e.g., Hartnett et al., 2017; Latimer, 2001; Van der Stouwe et al., 2014), and that the effectiveness of these interventions depends on the extent to which the principles of Risk, Need, and Responsivity for effective correctional treatment are adhered to (Dowden & Andrews, 2003). To successfully apply these principles in clinical practice, knowledge on the dynamic parenting factors most strongly associated with recidivism, as well as knowledge on the interrelatedness of these factors is essential. This study aimed to provide that knowledge by performing a network analysis of family and parenting risk factors for juvenile delinquency. Network analysis may reveal how these factors are interrelated and which factors are most “central” in a network, or in other words, are most strongly related to other factors in the risk factor network. These most central parenting factors can be important to address in family interventions for juvenile delinquency, because it may be expected that not only the central factors themselves improve, but also other related factors. Results of a network analysis may thus be used to strengthen family or parenting interventions for juvenile delinquency.

Juveniles engaging in criminal behavior often show multiple psychosocial needs that are rooted in ecological determinants of development (Snyder & Duchschere, 2022). Bronfenbrenner’s (1979; Bronfenbrenner & Morris, 2007) Ecological Systems Theory (EST) is very useful for understanding the complexity of these needs. The EST considers child development as the result of interactions between children and their environment, and describes an ongoing reciprocal interaction between a child and his social context that influences the way how a child develops. A child’s interactions with its social environment can occur on different social “subsystems” ranging from proximal interactions in the home environment with family members to more distal interactions within the cultural society a child is growing up in. In each of these social subsystems a variety of risk and protective factors may be present that influences a child’s behavior. These factors differ in the impact they have on behavioral outcomes in children: factors present in subsystems relatively close to the child (i.e., proximal factors) exert more influence than factors in subsystems further away from the child (i.e., distal factors). The immediate social environment of the child is in EST referred to as the microsystem and comprises individuals and social groups that are in direct contact with the child, such as family members, peers, school teachers, and classmates (e.g., Murray & Farrington, 2010). A child’s family is central to the microsystem, as it entails the primary context in which the child develops. Therefore, it can be expected that family and parenting factors are strongly predictive of juvenile delinquency.

The association between family functioning and juvenile delinquency has indeed been well documented in empirical literature (Assink et al., 2015; Datesman & Scarpitti, 1975; Glueck & Glueck, 1950; Howell & Hawkins, 1998: Manion & Wilson, 1995) with a stronger focus on the impact of family and parental risk factors than protective factors, presumably because the “bad is stronger than good” hypothesis (Baumeister et al., 2001) is dominant in the literature. Negative parent-child relationships in general and poor parenting skills in particular have been identified as significant risk factors for criminal behavior in youth (Lipsey & Derzon, 1998). For instance, poor parental supervision referring to a low level of parental monitoring of a child’s activities has been found to be a strong predictor of offending (Farrington & Loeber, 1999; Smith & Stern, 1997). That also holds for parental conflict and interparental violence: children experiencing parental conflict (Farrington & Loeber, 1999) and/or witnessing parental violence (Fergusson & Horwood, 1998) are far more likely to engage in offending behavior. A different and often used explanation for juvenile delinquency can be found in social learning theory suggesting that children’s behavior is shaped by parental rewards and punishments (Patterson, 1995). When parents do not respond in a consistent and contingent manner to their child’s antisocial and prosocial behavior, children tend to become more delinquent (Farrington, 2010). Another parenting behavior linked to juvenile delinquency with a strong empirical base is low parental support, with support referring to behavior that makes a child comfortable, accepted, and approved (Rollins & Thomas, 1979). Hoeve et al. (2009) showed in their meta-analysis that low levels of support—such as being neglectful, rejective, and hostile, are associated with juvenile delinquency, and more strongly than high levels of parental support. Finally, parental factors such as mental health problems and parental involvement in the criminal justice system have also been found to substantially increase the risk for juvenile delinquency (Lee, 2016; Moffitt & Caspi, 2001).

Given the strong ties between parental/family risk factors and juvenile delinquency, it is generally assumed that addressing family and parental problems in family interventions leads to a reduction of delinquent behavior in youth. These interventions are based on the therapeutic process that aims to modify psychological distress and/or problem behavior of family members by targeting individual problems and interpersonal relationships within the family. Although there is evidence that family interventions are effective in reducing recidivism in juvenile delinquents, multiple reviews of primary studies on the effects of family interventions on juvenile delinquency give a somewhat different picture. For instance, Latimer (2001) showed in his meta-analysis that intervention effects decrease substantially when the methodological quality of intervention evaluations is taken into account. Under the strictest methodological conditions, intervention effects even disappear. More recently, two reviews were conducted on family interventions that are commonly used in practice: Functional Family Therapy (FFT; Hartnett et al., 2017) and Multisystemic Therapy (MST; Van der Stouwe et al., 2014). Both reviews revealed that these interventions show only modest effects on juvenile delinquency.

The most influential theory on how treatment should be offered to effectively reduce the risk for future criminal behavior is the risk-need-responsivity (RNR) model (Andrews et al., 1990; Bonta & Andrews, 2010), which received empirical support in a substantial number of reviews (e.g., Dowden & Andrews, 1999a, 1999b, 2003; Koehler et al., 2012) although more convincing support is needed (Bijlsma et al., 2025). This model comprises three principles of which the need principle is particularly relevant for the current study. This principle answers the question *what* should be treated, and states that effective treatment should address criminogenic needs, which are changeable or dynamic risk factors that are (strongly) correlated with criminal conduct. Dowden and Andrews (2003) examined in their meta-analysis on family treatment for juvenile delinquency what role the RNR principles have in treatment effectiveness and found that family interventions are more effective when particularly the needs and responsivity principles are applied.

To successfully apply the RNR principles and the Needs principle in particular, it is essential to not only have knowledge on which dynamic family and parenting factors are (most strongly) associated with recidivism, but also to have insight into the interrelatedness of these factors. In studying interrelatedness in family risk factors for juvenile delinquency, we draw on the network theory of psychopathology that has been applied to different psychiatric disorders (Borsboom, 2017). In this theory, symptoms of disorders are assumed to play an active part in developing and maintaining psychopathology. By depicting psychopathology as a dynamic system, symptoms can play different roles in the development and maintenance of psychiatric disorders rather than being conceptually exchangeable (Blanken et al., 2018). Because of the potential co-occurrence and interrelatedness of symptoms, it is important to consider “propelling effects” from intervening on one symptom to other symptoms due to their potential interrelatedness (Lunansky et al., 2022). Put differently, a treatment targeting a specific symptom may alleviate other related symptoms as well. This network perspective can also be applied to juvenile delinquency and its risk factors: the risk for future criminal conduct increases as interrelated dynamic risk factors are activated and engage in feedback loops that trigger network activation. On the other hand, a risk reduction may occur by deactivating the risk factor network through addressing dynamic risk factors in treatment that weakens or dissolves the connection between those factors (cf. McNally, 2016).

Advances in methodology and statistics and the development of the network analysis technique have made it possible to study the complexity of the associations between risk factors for juvenile delinquency. Borsboom and Cramer (2013) were the first to apply network analysis to psychological attributes, and found that mental disorders result from a causal interplay between symptoms implying that the presence of a specific symptom causes another symptom to show up. In network analysis, the symptoms and their interrelations are presented in graphical networks in which the strength of the correlations between symptoms is determined after controlling for all other symptoms in the network (i.e., partial correlations; Epskamp, Waldorp, et al., 2018; Fisher et al., 2017; Van Borkulo et al., 2023). Besides examining interrelatedness between symptoms, a centrality analysis can be performed to examine which symptom (or symptoms) is most “central” in the network of all symptoms and is therefore most likely to cause the development or occurrence of other symptoms (Borsboom & Cramer, 2013). This centrality analysis provides important information on the treatment of disorders, as it can be expected that targeting the most central symptom or symptoms in treatment helps reducing other symptoms.

Network analysis has gained popularity in different research fields, such as clinical psychology, forensic psychology, psychiatry, personality research, social psychology, and quality of life research (Epskamp, Borsboom, & Fried, 2018). Recently, researchers showed that network analysis is also particularly useful in exploring how risk factors for a negative outcome influence each other, and in making inferences about the magnitude of these influences. For example, Van den Berg et al. (2020) studied how dynamic risk factors for sexual recidivism are interrelated in adult male sex offenders, and Vial et al. (2020) studied how risk factors for child maltreatment are interrelated and what factors are most important or urgent to address to reduce maltreatment risks.

This study is the first to explore how family and parenting risk factors for juvenile delinquency are interrelated by examining these factors from a network perspective. By doing so, more insight may be obtained into the family problems that are most “central” and thus essential or most urgent to address in juvenile delinquency treatment. Addressing these particular problems may alleviate related family and/or parenting factors as well. The results of network analysis may thus be used to strengthen family interventions for juvenile delinquency. A second aim was to examine whether the interrelatedness of parenting factors differs between counties, for instance because of societal and/or cultural differences. To do so, we chose to examine family risk factor networks in U.S. and Dutch families. The United States and the Netherlands show similarities such as valuing the individual, a multicultural society, a declining nuclear family ideal (i.e., father, mother, and two children), a growing number of single and newly formed families, and a diminished role of Church, but also differences such as different multiethnic profiles of the population, differences in access to health care, differences in identification and monitoring of mental health problems in school children, and differences in sentencing and rehabilitation (e.g., Breuk et al., 2006; Unger & De Paepe, 2019). Studying differences in interrelatedness between U.S. and Dutch families may illustrates whether cultural differences should be taken into account when deploying family interventions.

To meet both study aims, we conducted network analysis on data obtained from risk assessments of juvenile reoffending that were performed by probation officers in the United States and the Netherlands. Two networks capturing the interrelations between parenting factors were constructed: a U.S. sample network and a Dutch sample network. For both networks, we conducted centrality analyses to determine the factors that are most central in the risk factor network, and we examined differences in relations between—and centrality of—parenting risk factors across the two samples. In both samples, we also examined the prevalence of the parenting factors and the strength of the associations between the parenting factors and recidivism. We also tested differences in prevalences and associations between the two samples.

## Method

### Samples and Procedure

Secondary analyses were conducted on data that were previously collected with the Washington State Juvenile Court Assessment (WSJCA) in the United States and with a Dutch-adapted translation of the WSJCA in the Netherlands. Data were collected between July 2012 and January 2014 in a Dutch sample of juveniles, and between January 2000 and January 2001 in a U.S. sample of juveniles.

### U.S. Sample of Juveniles

The U.S. sample consisted of 13,613 juveniles from the United States that were 12 to 18 years old, and appeared in juvenile court because they were charged with a criminal offense and scored medium to high on the pre-screen of the Washington State Juvenile Court Assessment (WSJCA; see Instrument section). The sample consisted of 3,502 girls (26%) and 10,111 boys (74%). As for cultural background, 69% of the juveniles had a European American background, 11% had an African American background, 12% a Hispanic American background, and 8% had a different cultural background.

### Dutch Sample of Juveniles

The Dutch sample consisted of 3,630 juveniles from the Netherlands that were 12 to 18 years old, and appeared in juvenile court because they were charged with a criminal offense and scored medium to high on the Youth Actuarial Risk Assessment Tool (Y-ARAT; see Instrument section). The sample consisted of 410 girls (11%) and 3,220 boys (89%). Of all participants, 87% was born in the Netherlands and 13% was born outside the Netherlands.

### Selection of Family and Parenting Factors

The WSJCA is a screening and risk assessment instrument that was developed in Washington State (Barnoski, 2004). The WSJCA maps out the most important risk and protection factors for a large number of life domains. The choice of factors being assessed in the WSJCA was driven by a review of the juvenile delinquency research literature and feedback from an international team of experts. The initially selected items were revised again following critical evaluations of Washington State juvenile court professionals (Barnoski, 2004). The WSJCA comprises a prescreen that is relatively short in length and a full assessment. The prescreen assessment is administered in all juveniles that are on probation and is a shortened version of the full assessment. It indicates in a quick manner whether a juvenile has a low, moderate, or high risk to reoffend. In Dutch juveniles, the Y-ARAT (Van der Put, 2014) is used for this purpose. The WSJCA full assessment is only administered in youth that are assessed with the WSJCA prescreen (U.S. juveniles) or Y-ARAT (Dutch juveniles) as having a moderate or high risk for reoffending. The WSJCA full assessment identifies a juvenile’s risk and protective factors profile to guide rehabilitative efforts. For this study, we selected the dynamic parenting factors of the family domain. Table 1 shows these parenting variables and their response categories.

**Table 1.** Family Risk Factors and Their Scoring in the (Dutch-Translated) WSJCA That Was Administered in the U.S. and Dutch Sample of Juveniles.

Family/parental risk factors (in bold) with their WSJCA items	Response categories and scoring
**Lack of family support (1)** Family willingness to help support youth	0 = Consistently willing to support youth 1 = Inconsistently willing to support youth 2 = Little or no willingness to support youth
**Family provides no opportunities (2)** Family provides opportunities for youth to participate in family activities and decisions affecting the youth	0 = Opportunities for involvement provided 1 = Some opportunities for involvement provided 2 = No opportunities for involvement provided
**Serious conflicts (3)** Level of conflict between parents, between youth and parents, among siblings	0 = Some conflict that is well managed 1 = Verbal intimidation, yelling, heated arguments 2 = Threats of physical abuse
**Lack of parental supervision (4)** Parents know whom youth is with, when youth will return, where youth is going, and what youth is doing.	0 = Consistent good supervision 1 = Sporadic supervision 2 = Inadequate supervision
**Inadequate parental punishment (5)** Consistent appropriate punishment for bad behavior	0 = Consistently appropriate punishment 1 = Consistently overly severe punishment 2 = Inconsistent or erratic punishment
**Inadequate parental reward (6)** Consistent appropriate rewards for good behavior	0 = Consistently appropriate rewards 1 = Consistently overly indulgent/overly protective, 2 = Inconsistent or erratic rewards
**No parental rejection of antisocial behavior (7)** Parental characterization of youth’s anti-social behavior	0 = Disapproves of youth’s anti-social behavior, 1 = Minimizes, denies, justifies, excuses behavior, or blames others/circumstances 2 = Accepts youth’s anti-social behavior as okay

*Note.* The numbers in parentheses refer to the node numbers in the networks (See Figures 1 and 2) and the risk factor numbers in Tables 4 and 5. The seven WSJCA items and their response categories presented here are described in the WSJCA manual (Barnoski, 2004).

The WSJCA is administered by probation officers who are trained in assessing juveniles by certified trainers. Part of the training is a review of multiple video-taped interviews and the assessment following each interview to ensure that probation officers have mastered the required assessment skills. The family factors examined in this study were measured over a period of 6 months prior to the date of the WSJCA assessment, so that the family risk factors were present at the time of the assessment or shortly before the moment of assessment (6 months at most). All questions were asked to both the juvenile and his/her family members. If conflicting answers were given by family members, the probation officer weighted the answers on their accuracy and selected the most appropriate response. This selection could be based merely on the clinical experience and knowledge of the officer, or on additional information that was collected by the officer, for instance by consulting school or other professionals who were in regular contact with the juvenile.

### Operationalization of Recidivism

Recidivism was defined as the occurrence of one or more new convictions within 18 months after the WSJCA was administered. Recidivism data were obtained from the national governmental database of officially recorded convictions. The data were retrieved by the authors of this study in anonymous form, as the WSJCA scores and the recidivism rate of each offender in the sample were merged with an anonymous identifier, meaning that data could not be linked to identifiable or individual offenders. As for the Dutch sample, only the probation services could link recidivism data to individual offenders with an anonymous identifier. To adequately measure 18-month recidivism, a period of 30 months was required: an 18-month re-offending follow-up period and another 12-month period to allow for any re-offenses to be adjudicated. Recidivism was treated as a dichotomous variable (whether or not a juvenile was convicted for any new offense).

### Statistical Analyses

SPSS Statistics version 28 (IBM Corporation, 2021) was used to calculate prevalences of family risk factors and Pearson’s correlations for the association between family risk factors and recidivism. In addition, SPSS was used for testing differences in risk factor prevalences between U.S. and Dutch youth. The Jeffrey’s Amazing Statistics Program (JASP, version 0.16, JASP Team, 2021) was used to conduct the network analyses.

To analyze the interrelatedness in family risk factors for juvenile delinquency, a visualized network analysis was performed in JASP. The networks were estimated with the Graphical Least Absolute Shrinkage and Selection Operator (GLASSO) algorithm (Friedman et al., 2014) that was based on the Extended Bayesian Information Criterion (EBIC; Chen & Chen, 2008). GLASSO provides a parsimonious network (i.e., fewer connections between risk factors) in which the most important empirical associations are represented (Epskamp, Borsboom, & Fried, 2018). EBIC was used to obtain the optimal estimate of the partial correlation matrix aligned with the tuning parameter (Friedman et al., 2014). EBIC is the most conservative option for penalization and the most suitable option for categorical data (Haslbeck & Waldorp, 2020). The tuning parameter was set to 0.5 to obtain a parsimonious network and to facilitate interpretation of the network (i.e., fewer associations in the network and higher specificity and sensitivity).

Each “node” of a network represents a family risk factor for recidivism. Associations between risk factors—while controlling for all other risk factors in a network—are represented by solid lines in the graphical network that are referred to as “edges.” The thickness of these edges indicates the strength of an association with thicker edges representing stronger associations. Blue edges represent positive associations and red edges represent negative associations between network nodes. To make inferences about the importance of each risk factor (or “node”) in the network, the “node strength centrality” was examined, as this centrality type is the most stable of the three centrality measures (i.e., strength, betweenness, and closeness) (Epskamp, van Borkulo et al., 2018). This centrality measure captures the absolute sum of all associations each node has with the other network nodes, and provides an indication of how strong a node’s connections are with all other nodes (Fried et al., 2016; Opsahl et al., 2010). Node strength centrality coefficients were standardized to enable comparison of this centrality measure across networks. Two networks were obtained in JASP, one for the U.S. sample of juveniles and one for the Dutch sample of juveniles.

After constructing the networks, we determined the node centrality stability of each network by calculating correlation stability (CS) coefficients after taking 500 bootstrap samples from the original U.S. and Dutch samples of juveniles. Node centrality is considered to be stable when CS coefficients exceed the value of 0.25, although values higher than 0.50 are to be preferred (Epskamp, van Borkulo et al., 2018). The edge weight accuracy was assessed by calculating and inspecting confidence intervals using non-parametric bootstrapping (500 samples).

### Ethical Approval

Formal Institutional Review Board (IRB) approval for conducting this study was not required, as it concerned analyses of secondary and already de-identified data. As such, this study does not pose harm to study subjects, and therefore did not require formal IRB approval. This study was conducted in accordance with the rules and guidelines of the Faculty Ethics Review Board (FMG-UvA) of the University of Amsterdam, the Netherlands.

## Results

Table 2 shows the extent to which the various family and parenting risk factors occur, separately for the U.S. and Dutch sample. Juveniles in the United States had a higher risk score than Dutch juveniles for all risk factors, except for “serious conflicts,” for which Dutch youth had a higher risk score than U.S. youth. Table 3 shows the strength of the associations between the risk factors and recidivism, separately for the U.S. and Dutch sample. Most factors were significantly related to recidivism in both samples, although effect sizes were (very) small in magnitude. There were no significant differences between U.S. and Dutch juveniles in the strength of the associations between the parenting factors and recidivism.

**Table 2.** Prevalence of Family Risk Factors in the U.S. and Dutch Samples of Juveniles (Mean Values of WSJCA Item Scores).

	U.S. juveniles (*N* = 13,613)	Dutch juveniles (*N* = 3,630)	*F*
Lack of family support	0.47	0.28	262.84*
Family provides no opportunities	0.93	0.52	1,159.25*
Serious conflicts	0.87	1.06	152.29*
Lack of parental supervision	0.76	0.52	297.89*
Inadequate parental punishment	0.78	0.61	121.70*
Inadequate parental reward	0.76	0.29	794.05*
No parental rejection of antisocial behavior	0.24	0.38	168.04*

* *p* < .01.

**Table 3.** Associations (Pearson’s *r*) Between Family Risk Factors and Recidivism.

	U.S. youth (*N* = 13,613)	Dutch youth (*N* = 3,630)	*z*
Lack of family support	.06*	.05*	0.59
Family provides no opportunities	.07*	.08*	‒0.54
Serious conflict	.11*	.08*	1.67
Lack of parental supervision	.10*	.13*	‒1.25
Inadequate parental punishment	.08*	.06*	1.40
Inadequate parental reward	.08*	.05*	1.50
No parental rejection of antisocial behavior	.01	.03	‒1.12

*Note.* Differences between (independent) correlations were tested with a *z* test of which none were significant.

* *p* < .01.

**Figure 1.**
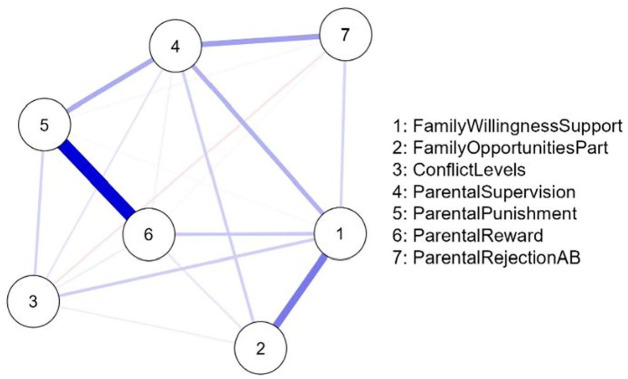
EBICGlasso network for the U.S. sample (*N* = 13,613) showing the interrelations between the parenting factors. *Note.* Most associations were positive and represented by blue lines. Two associations were negative and represented by red lines. The factors “parental supervision” (#4) and “parental punishment” (#5) were most central, whereas the factor “serious conflict” (#3) was the least central. See Table 1 for risk factor details.

**Figure 2.**
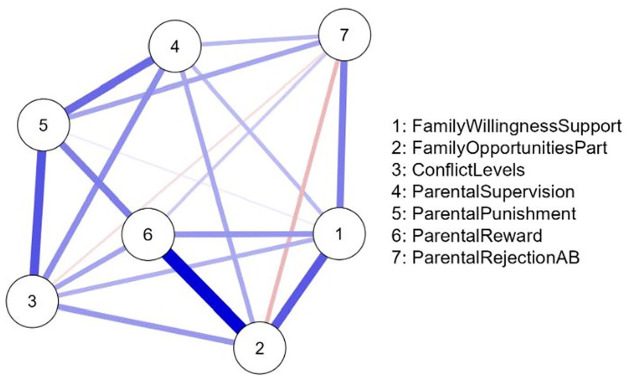
EBICGlasso network for the Dutch sample (*N* = 3,630) showing the interrelations between the parenting factors. *Note*. Most associations were positive (blue lines), but two associations were negative (red lines). The factors “family provides no opportunities” (#2) and “inadequate parental reward” (#6) were most central, whereas the risk factor “lack of parental supervision” (#4) was the least central. See Table 1 for risk factor details.

The network of parenting risk factors for the U.S. sample is shown in Figure 1 and for the Dutch sample in Figure 2. The networks consisted of 7 nodes and 20 non-zero “edges.” Each node represents a parenting factor, and each edge (the line between two nodes) represents a partial correlation between two parental factors controlling for all other factors in the network. The thickness of the edges represents the strength of the (partial) correlation between two risk factors controlling for all other risk factors in the network. Table 4 shows the weights matrix of all partial correlations in the network that are represented by the lines in Figures 1 and 2. As expected, almost all associations between the family risk factors are positive in both the U.S. sample and Dutch sample networks. However, in U.S. juveniles, the network reveals a small but negative association between “parental rejection of antisocial behavior” and “serious conflicts,” and between “parental punishment” and “family provides no opportunities.” In Dutch juveniles, we found that “parental rejection of antisocial behavior” is negatively associated with “family provides no opportunities” and “serious conflicts,” although these associations were small in magnitude.

**Table 4.** Weights Matrix with Partial Correlations Between Family Risk Factors for U.S. Juveniles (Below Diagonal) and Dutch Juveniles (Above Diagonal).

Family risk factor	1	2	3	4	5	6	7
Family willingness support (1)	—	0.26	0.12	0.11	0.03	0.17	0.21
Family opportunities part (2)	0.39	—	0.16	0.14	0.00	0.40	*−0.12*
Conflict levels (3)	0.14	0.05	—	0.18	0.27	0.14	*−0.05*
Parental supervision (4)	0.22	0.13	0.07	—	0.24	0.00	0.11
Parental punishment (5)	0.02	*−0.01*	0.12	0.24	—	0.20	0.14
Parental reward (6)	0.15	0.07	0.05	0.04	0.74	—	0.08
Parental rejection AB (7)	0.12	0.00	*−0.07*	0.28	0.03	0.02	—

*Note*. Italicized values represent negative associations between factors (while controlling for all other associations in the network). A minimum absolute value of 0.03 is considered worthy of interpretation (Isvoranu et al., 2017). The family risk factor labels correspond with the labels in the graphical networks in Figures 1 and 2 (see Table 1 for risk factor details).

The mean of the edge weights (i.e., partial correlations) was higher for the Dutch sample than for the U.S. sample, and thus the partial correlations are in general stronger in the Dutch sample. In both networks, there is a strong association between the factors “parental punishment” and “parental reward.” This association is stronger for United States than for Dutch juveniles and is the strongest association in the network of U.S. juveniles. In the Dutch sample, the strongest association in the network is between the factors “family provides no opportunities” and “inadequate parental reward,” whereas in the U.S. sample these factors are not associated.

**Figure 3.**
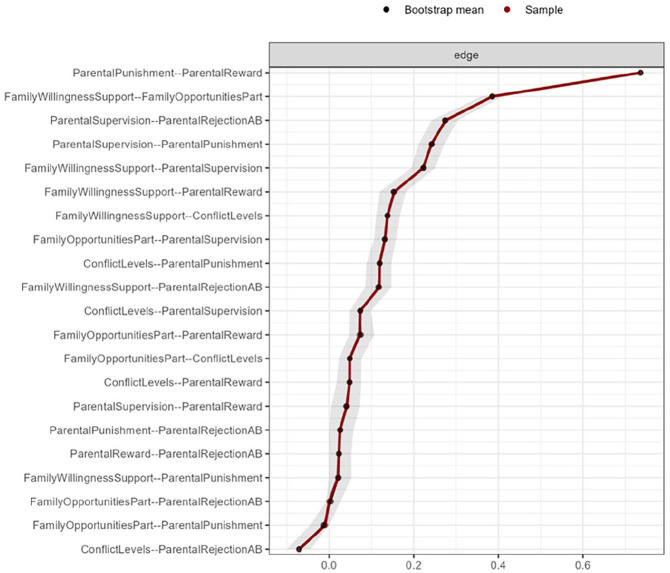
Edge stability estimate in the U.S. sample of juveniles using non-parametric bootstrapping. *Note.* The *x*-axis represents the edge strength, while specific edges (individual associations) are represented on the *y*-axis. The red line shows the estimate of the edge stability, and the gray bars the corresponding 95% confidence intervals. Sample and bootstrap estimates almost fall together, and therefore only very minimal differences can be seen in this plot.

**Figure 4.**
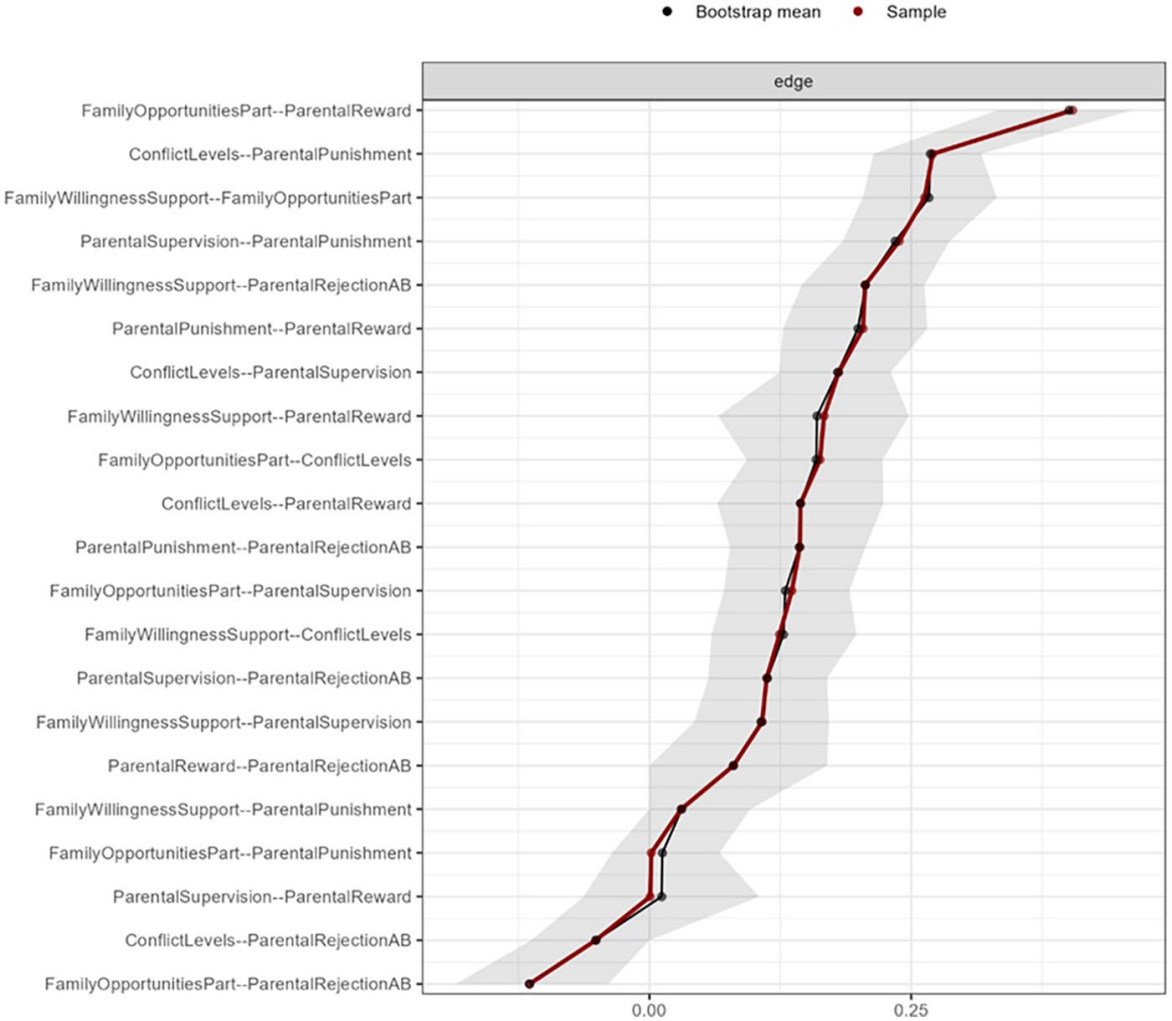
Edge stability estimate in the Dutch sample of juveniles using non-parametric bootstrapping. *Note.* The *x*-axis represents the edge strength, while specific edges (individual associations) are represented on the y-axis. The red line shows the estimate of the edge stability, and the gray bars the corresponding 95% confidence intervals. Sample and bootstrap estimates almost fall together, but some differences between the curves can be observed.

Both the U.S. and Dutch sample networks were sufficiently stable. In the U.S. sample network, the CS coefficients of the node strength centrality ranged from 0.5 to 1.2 for the seven parenting factors and were all above the minimum threshold of 0.25 and the preferred threshold of 0.50 for stability of coefficients (Epskamp et al., 2018). The edge stability plot (see Figure 3) reveals that the confidence interval around 5 of the 21 estimated edges contains zero and that most intervals did not contain zero. Also, the confidence intervals are rather small indicating that a substantial number of edges differ significantly from each other. This implies that the edge weights can be interpreted with substantial confidence. In the Dutch sample network, the CS coefficients of the node strength centrality ranged from 0.71 to 1.1 and were all above the preferred threshold of 0.50. The edge stability plot (see Figure 4) once again reveals that the confidence interval around 5 of the 21 estimated edges contains zero while most intervals did not contain zero. The intervals are wider compared to the intervals in the U.S. sample indicating that less edges than in the U.S. sample differ significantly from each other. Therefore, interpretation of the edge weights can be performed with moderate confidence, and that more caution is advised in ordering the edge weights compared to the U.S. sample.

**Figure 5.**
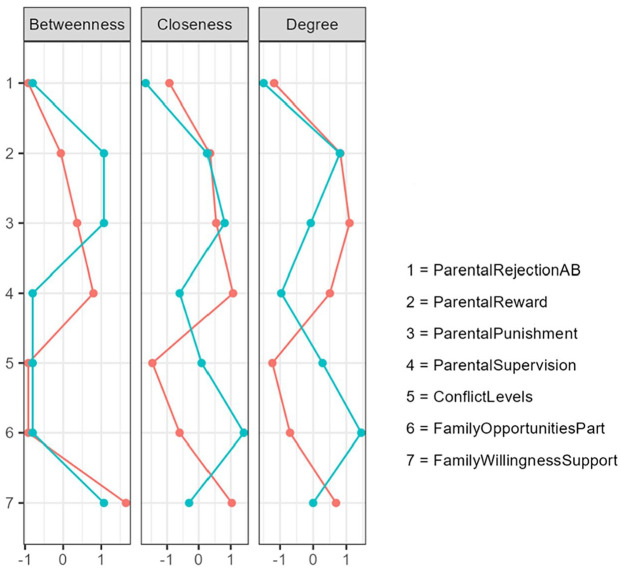
Centrality plots for the U.S. (red line) and Dutch sample networks illustrating betweenness, closeness, and degree (strength) of each of the seven family risk factors. *Note.* Centrality indices are standardized *Z* values (*X*-axis) with the red line representing the U.S. sample and the green line representing the Dutch sample. Betweenness represents the degree of connectivity of factors; Closeness represents the distance centrality of factors; Strength represents the degree centrality or node strength centrality of factors, and is the most stable of the three centrality measures (see Methods section). Higher strength indicates greater overall importance to the network. See Table 1 for risk factor details.

The betweenness, closeness, and strength (“degree”) of each parenting factor are presented in Table 5 and Figure 5 for both the U.S. and Dutch sample networks. All centrality indices are standardized *Z* scores and provide relative importance of the factors in the network. In the U.S. network, “inadequate parental punishment” and “lack of parental supervision” were most central, whereas in Dutch youth, “inadequate parental reward” and “family provides opportunities” were most central.

**Table 5.** Centrality of the Family Risk Factors in the U.S. and Dutch Sample.

Family risk factor	U.S. sample			Dutch sample		
	Betweenness	Closeness	Strength	Betweenness	Closeness	Strength
Family willingness support (1)	1.65	1.03	0.69	1.07	−0.30	−0.002
Family opportunities part (2)	−0.92	−0.60	−0.70	−0.80	1.42	1.45
Conflict levels (3)	−0.92	−1.47	−1.23	−0.80	0.10	0.28
Parental supervision (4)	0.80	1.07	0.50	−0.80	−0.60	−0.96
Parental punishment (5)	0.37	0.54	1.10	1.07	0.81	−0.07
Parental reward (6)	−0.06	0.35	0.82	1.07	0.27	0.81
Parental rejection AB (7)	−0.92	−0.93	−1.18	−0.80	−1.68	−1.50

*Note.* Centrality indices are standardized *Z* values. Betweenness represents the degree of connectivity of factors; Closeness represents the distance centrality of factors; Strength represents the degree centrality or node strength centrality of factors, and is the most stable of the three centrality measures (see Methods section). Higher values indicate more importance to the network. The family risk factor labels correspond with the labels in the graphical networks in Figures 1 and 2 (see Table 1 for risk factor details).

## Discussion

This study examined the strength of the association between several parenting factors and juvenile re-offending, how these factors are interrelated, and whether the associations and interrelatedness differ between juveniles (and their family members) from the Unites States and the Netherlands. Despite the numerous studies examining the relationship between parenting factors and criminal recidivism, few studies examined the interrelatedness of parenting factors, while this knowledge may inform attempts to strengthen family interventions for juvenile delinquency. This study was the first to examine family and parenting risk factors for juvenile delinquency from a network approach, and as such the first to apply network analysis to study the complexity of the associations between these factors.

The results showed that most parental risk factors were significantly related to recidivism in both the U.S. and Dutch sample, which is in line with previous literature (e.g., Farrington, 2010; Farrington & Loeber, 1999; Flanagan et al., 2019; Hoeve et al., 2009; Williams & Smalls, 2015), although the identified associations were rather weak in strength. Parental supervision was the only factor with a correlation stronger than the lower bound (*r* = .10) for a small association (Cohen, 1992) in both the U.S. and Dutch sample of juveniles. These rather low factor impacts may not be surprising as we know from previous work that the impact of dynamic risk factors on recidivism decreases as children grow older and become adolescents (Van der Put et al., 2010, 2011). Moreover, criminal behavior is the result of complex interactions between different types of risk factors (Prinzie et al., 2008). This implies that re-offending is not so much predicted by individual risk factors with a relatively low impact, but by the presence of multiple risk factors (Loeber et al., 2009) of which the cumulative effect is substantially stronger than the effect of individual risk factors. We did not find a significant association between parental rejection toward antisocial behavior and recidivism in any of the two samples, which might indicate that parental attitudes do not play a role in juvenile delinquency. However, previous research has revealed that parents holding *favorable* beliefs toward antisocial behavior is a substantial risk factor for juvenile delinquency (e.g., Maguire & Fishbein, 2016). It may be that the parental rejection item in the WSJCA should be phrased differently to better capture the role of parental attitudes toward antisocial behavior.

Network analyses showed that in the U.S. sample network, “inadequate parental punishment” and “lack of parental supervision” were most central, whereas in the Dutch network, the most central factors were “family provides no opportunities” and “inadequate parental reward.” The analyses thus seem to reveal differences in risk factor dynamics between U.S. and Dutch juveniles and their families. The most central factors in the U.S. sample network refer to aspects of an authoritarian parenting style, whereas in the Dutch sample network, the most central factors refer to aspects of an authoritative parenting style. An authoritarian parenting style encompasses adult-oriented, coercive, restrictive, and firm discipline techniques and emphasizes the negative aspects of control such as harsh punishment and love withdrawal, whereas an authoritative parenting style encompasses child-oriented and inductive discipline techniques such as guiding the child’s behavior cognitively, providing the child information and knowledge, and stimulating responsible behavior of the child (e.g., Baumrind, 1968, 1971). In general, authoritative control positively affects child behavior and development, while authoritarian control has been found to negatively affects child behavior and development (e.g., Baumrind, 1966). A strict authoritarian control (Farrington, 1989) and harsh punishment (Farrington et al., 2003) are associated with high levels of delinquent and antisocial behavior, although effect sizes vary substantially across studies (Loeber & Stouthamer-Loeber, 1986).

Some limitations of this study need to be mentioned. First, the WSCJA was not designed to provide an in-depth examination of risk factors. Instead, this risk assessment instrument is designed to be used by juvenile justice professionals and clinicians to summarize juveniles’ risks and needs, classify their overall risk level, and plan treatment and supervision strategies. Second, the examined risk factors were measured with single items which limits the ability to assess multiple facets of a construct. For instance, family support is broadly assessed with one item that specifically assesses a family’s willingness to support a juvenile, but parents can only be supportive when they are sensitive to the emotional, cognitive, and social needs of their child which are not assessed in the WSJCA. A more thorough assessment of the family and parental factors may better capture the interrelatedness between the factors, although most studies on single-item assessments generally reveal that they are just as valid and reliable as their multi-item counterparts (Ahmad et al., 2014; Ang & Eisend, 2018). Third, the sample predominantly consisted of moderate- and high-risk youth. Therefore, the results cannot be generalized to juvenile delinquents with lower risks of recidivism. Finally, recidivism was assessed with official records of recidivism (i.e., registered convictions) that suffer from the risk of underestimating, as the registered number of criminal acts in official records is lower than the actual number of committed offenses. Therefore, convictions represent a conservative estimate of reoffending.

Applying a network approach to studying behavior has gained much attention in recent years. More and more researchers acknowledge that different types of behavior can be conceptualized as a complex dynamic system comprising different types of factors or symptoms that influence each other. As such, an increasing number of studies in which network analysis is performed has emerged. For instance, studies on the interrelatedness of resilience factors for substance abuse symptoms (Rhemtulla et al., 2016), the interrelatedness of risk factors for child maltreatment (Vial et al., 2020), the interrelatedness of risk and vulnerability factors for radicalization (Clemmow et al., 2023), and the interrelatedness of dynamic risk factors for sexual recidivism in adult male sex offenders (Van den Berg et al., 2020). Also in studying parental risk factors for criminal recidivism in juveniles, the network framework provides opportunities to increase our understanding of how different factors interact and play a role in the development and maintenance of criminal behavior. The current study provides further insight into the interrelatedness of parenting risk factors for juvenile re-offending and underline that analyzing risk factors in a psychometric network modeling approach is a promising direction for future research.

The present study provides support for conceptualizing risk factors for criminal recidivism in juveniles as a dynamic system. Although we generally found stronger associations in the Dutch sample of juveniles than the U.S. sample, the networks for both samples revealed an interrelatedness in risk factors that stress the complexity of criminal behavior and the underlying risk factors that influence each other. Additionally, our results provide first indications that some parenting risk factors may be “stronger” than others, implying there are differences across parenting factors in the importance they have in a network of interdependent risk factors. The parenting factors identified in this study as most central may be informative for attempts to strengthen family interventions for juvenile delinquency. It may be expected that targeting central risk factors will not only reduce the parenting problems they represent, but also related parenting problems. Put differently, targeting the central factors may induce the most change in the network of risk factors, which may break up the network if effective treatment is offered to juveniles and their family members. Our study also illustrates that analyzing risk factors in a psychometric network approach yields a rather simple and easily understandable model of a complex and multifactorial behavioral problem, and offers insights into how clinical practice can be improved. Therefore, network analysis proves to be an important technique with vast opportunities for future research aimed at increasing our knowledge of the etiology of juvenile delinquency and for research aimed at strengthening interventions for juvenile delinquency.
